# Comparative Assessment of Vitamin-B12, Folic Acid and Homocysteine Levels in Relation to p53 Expression in Megaloblastic Anemia

**DOI:** 10.1371/journal.pone.0164559

**Published:** 2016-10-25

**Authors:** Manish K. Yadav, Nandini M. Manoli, SubbaRao V. Madhunapantula

**Affiliations:** 1 Department of Pathology, JSS Medical College, JSS University, Mysuru - 570015, Karnataka, India; 2 Center of Excellence in Molecular Biology and Regenerative Medicine (CEMR), Department of Biochemistry, JSS Medical College, JSS University, Mysuru - 570015, Karnataka, India; Universitat des Saarlandes, GERMANY

## Abstract

**Background:**

Megaloblastic anemia (MBA), also known as macrocytic anemia, is a type of anemia characterized by decreased number of RBCs as well as the presence of unusually large, abnormal and poorly developed erythrocytes (megaloblasts), which fail to enter blood circulation due to their larger size. Lack of vitamin-B12 (VB12) and / or folate (Vitamin-B9, VB9) with elevated homocysteine is the key factor responsible for megaloblastic anemia. Prior studies have demonstrated the induction of apoptosis in these abnormal under-developed erythrocytes. However, it is not clear whether this apoptosis induction is due to elevated p53 level or due to any other mechanism. Furthermore, it is also not fully known whether decreased vitamin-B12 and / or folate are responsible for apoptosis induction mediated by p53 in pre-erythroblasts.

**Methods:**

Levels of serum VB9, VB12 and homocysteine in 50 patients suffering from MBA were compared with 50 non-megaloblastic anemia control subjects, who were referred by the clinicians for bone marrow examination for medical conditions other than MBA. Next, we have measured the p53 expression in the paraffin embedded blocks prepared from bone marrow biopsy, using immunohistochemistry, and the expression levels correlated with VB9 and VB12 levels.

**Results:**

Out of 50 MBA patients 40 (80%) and 44 (88%) subjects had very low VB12 and VB9 levels respectively. In contrast, only 2 (4%) and 12 (24%) non-megaloblastic anemia controls, out of 50 subjects, had low VB12 and VB9 respectively. Correlating with low vitamin B9 and B12, the homocysteine levels were high in 80% cases. But, only 20% non-megaloblastic controls exhibited high homocysteine in plasma. Immunohistochemical analysis for p53 expression showed a significantly high level of expression in MBA cases and no—or very low—expression in control subjects. Our correlation studies comparing the VB12 and VB9 levels with p53 expression concludes unusually high p53 levels in patients suffering from VB12 and VB9 deficiency induced MBA compared to control subjects not suffering from MBA.

**Conclusion:**

Tumor protein p53 is the key protein expressed heavily in the bone marrow biopsies of patients suffering from VB12 and VB9 deficiency induced MBA but not in control subjects. Hence, p53 expression could be used as a surrogate marker for confirming the VB9 and VB12 induced MBA.

## Background

Megaloblastic anemia (MBA), also known as macrocytic anemia, is characterized by decreased number of RBCs as well as the presence of unusually large, abnormal and poorly developed erythrocytes (megaloblasts), which fail to enter blood circulation due to their larger size [1]. MBA is caused by low vitamin B12 (VB12, Cyanocobalamin) and / or decreased Vitamin B9 (VB9, Folate) [2]. VB12 deficient megaloblastic anemia (MBA), referred as pernicious anemia, is caused either by lack of (a) sufficient amount of VB12 in diet and / or (b) intrinsic factor responsible for VB12 absorption [3]. MBA is the primary cause for damage to neurons, digestive disorders, weekend bones, and even to stomach cancer. Since majority of individuals suffering from MBA stay asymptomatic for many years and exhibit characteristic features such as pale skin color (Pallor), shortness and difficulty of breathing, light-headedness and fatigue only at later stages, early diagnosis is difficult [1]. Furthermore, individuals with MBA exhibit tingling and numbness of hands and feet in the initial stages, but once advanced, exhibit symptoms such as loss of memory and vision [4,5]. This loss of memory is due to increase in the homocysteine (HCys) levels in serum [6]. As increased homocysteine is also known to induce atherosclerosis, dementia and Alzheimer’s disease, timely diagnosis and therapeutic interventions are very important for better treatment outcomes [7,8]).

Mechanistically, the MBA caused by Folate or Vitamin-B12 deficiency is due to the inhibition of DNA synthesis and induction of apoptosis in pre-erythroblasts, which hinders the transformation of these cells in to reticulocytes thereby ultimately leading to pancytopenia. Elevated apoptosis in pre-erythroblasts is primarily caused by the inability of these cells to repair the DNA damage as well as improper replication [9,10]. Molecularly, Folate and Vitamin-B12 deficiencies limit the levels of Tetrahydrofolate (THF), which hinders the synthesis of thymidylate, purines and methylation of cytosine residues [11]. For example, conversion of dUMP in to dTMP is inhibited by the loss of BV9 and VB12, leading to the accumulation of dUMP in the DNA causing double strand breaks [12,13]. Cells containing nicked DNA either rectify the damage by DNA-repair processes or undergo apoptosis to prevent the accumulation of degraded DNA [14]. One key apoptosis inducer is p53 [15]. It is well-established that in addition to the induction of apoptosis in abnormal cells, p53 has a role in the terminal differentiation of normoblasts and maintain the integrity of genetic material by protecting the DNA from various damages as well as from mis-incorporation of nucleotides [16,17,18]. However, controversial findings have been reported about the role of p53 in apoptosis induction in megaloblast cells. While one study reported p53-dependent apoptosis, the other study demonstrated p53-independent apoptosis [19,20]. Therefore, to address whether low VB12 and VB9, confirmed by elevated homocysteine content, correlates with the expression of p53 in megaloblastic anemia, we have measured the levels of vitamin-B9, B12 and homocysteine in the serum of non-MBA control subjects and MBA patients, and quantified the p53 expression using immunohistochemistry of bone marrow biopsies embedded in paraffin blocks. The data showed elevated expression of p53 in all the MBA samples indicating that p53 is the primary cause for the induction of apoptosis in megaloblast cells isolated from pernicious anemia patients.

## Materials and Methods

### Screening and selection of megaloblastic anemia cases and non-megaloblastic anemia control subjects

The study was approved by Institutional Ethical Committee (Jagadguru Sri Shivarathreeshwara / Medical College / Institutional Ethical Committee /MC/IEC/1847/2012-2013; Dated 21^st^ July 2012), which is according to the principles of declaration of Helsinki. Informed written consent approved by the Jagadguru Sri Shivarathreeshwara / Medical College / Institutional Ethical Committee /MC/IEC/1847/2012-2013;Dated 21^st^ July 2012 was taken from each individual after explaining the study details in their native language.

**Fig 1:**
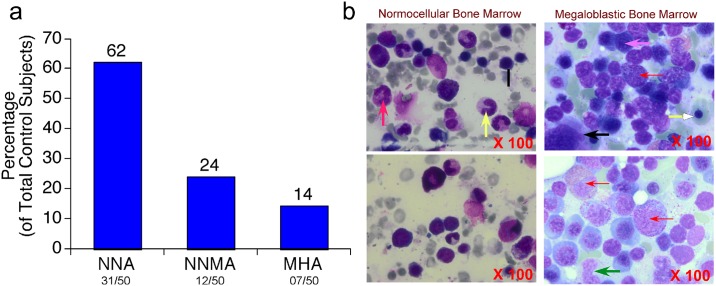
Distribution of control non-megaloblastic anemia participants & Analysis of bone marrow collected from non-megaloblastic anemia controls and megaloblastic anemia cases using Leishman’s staining. (a) Analysis of non-megaloblastic anemia control subjects using bone marrow aspirate normocytic-normochromic anemia (62%, 31/50); normocytic-normochromic with microcytic hypochromic anemia (24%, 12/50) and microcytic hypochromic anemia (14%, 7/50) (b) Photomicrographs (at 100X magnification) of Leishman’s stained bone marrow samples collected from control non-megaloblastic anemia and cases with megaloblastic anemia. Bone marrow from control subjects showed normal cellularity with erythroid cells showing—normal maturation (Black arrow),—myeloid series cells and megakaryocytes along with mature neutrophils (Red arrow) and Band forms (Yellow arrow). Megaloblastic anemia cases showed erythroid hyperplastic cells (Pink arrow) and open chromatin (Red arrow). Cells with more cytoplasm (Yellow arrow), giant sized nuclei (metamyelocytes, Black arrow) and band forms (neutrophils, Green arrow) and nuclear hyper segmentation and abnormal maturation (megakaryocytes) were observed only in MBA cases but not in control subjects.

Identification of non-megaloblastic controls: The control non-megaloblastic subjects have been selected by clinical diagnosis of patients for suspected anemia with symptoms of weakness, fatigue and tiredness with fever. These subjects are screened by at least three experienced pathologists for the changes in: (a) peripheral blood smear; (b) bone marrow aspirate, and (c) biopsy. In addition, hematological parameters and biochemical parameters (Folate and VitB12) were also assessed while selecting non-megaloblastic control subjects. Based on the above analysis the control subjects are grouped in to: (a) normocytic-normochromic anemia (62%, 31/50); (b) normocytic-normochromic with microcytic hypochromic anemia (24%, 12/50) and (c) microcytic hypochromic anemia (14%, 7/50) (Fig 1a; Tables A, B and C in S1 File). The normocytic normochromic anemia patients were characterized by hemolysis or hemorrhage with normal MCV, MCH and MCHC but low RBC count and hematocrit. The microcytic anemia patients were characterized by small hypochromic red blood cells in a peripheral blood smear with a low MCV (less than 83micron).

Identification of megaloblastic anemia cases: Megaloblastic anemia cases were identified by examining the individuals for characteristic clinical features such as anemia and the symptoms that include glossitis, weakness, fatigue, pallor and neurological manifestations [21,22]. In addition, the selected individuals were subjected to peripheral blood smear reporting that consist of macrocytosis, macro-ovalocytes, basophilic stippling, giants platelet forms, hyper segmented neutrophils, mean corpuscular volume > 100fL [23]. Further, bone marrow aspirate reporting assessing the hyper-cellular bone marrow for erythroid hyperplasia with megaloblasts, giant metamyelocytes band forms, giant megakaryocytes was also considered for recruiting the subjects in this study (Fig 1b). Selected cases were further analyzed for the levels of Vitamin-B9 and B12 using Beckman Coulter Unicel DXI600, and Homocysteine using Randox BIO -02 RX Imola Chemistry Analyzer (Model# OPT790DT). Complete blood count (CBC) in EDTA blood samples were analyzed using Sysmex—XN– 1000 Automated Hematology Analyzer. In brief, ~3ml blood was collected from megaloblastic bone marrow cases and non-megaloblastic control patients in to EDTA containing vacutainer tube and the plasma was separated by centrifugation at 3000rpm in a laboratory centrifuge. The separated plasma was stored at -80°C immediately.

#### Estimation of Vitamin-B12

For analysis of VB12, 10μl plasma was subjected to an automated chemiluminescent two step competitive Immunoassay as described in the product manual [24]. Experimentally, the plasma samples were incubated with mouse anti-Vitamin B12 binding protein, followed by the addition of Vitamin-B12 alkaline phosphatase conjugate and goat anti-mouse capture antibody coupled to paramagnetic particles. After incubation, the chemiluminescent substrate Lumi-phos* 530 was added to measure Vitamin-B12 levels using a luminometer.

#### Estimation of Folic Acid

The folic acid levels in EDTA plasma sample was carried out using a quantitative automated two-step competitive immunoassay method, which uses a mouse anti-Folic Acid binding protein, Folic Acid alkaline phosphatase conjugate and goat anti-mouse capture antibody coupled to paramagnetic particles. The chemiluminescent substrate Lumi-phos* 530 is added to the vessel and the light generated by the reaction was measured with a luminometer.

#### Estimation of Homocysteine

The homocysteine concentration in EDTA plasma samples was carried out by enzymatic method using homocysteine estimation kit (Proton PB-HCY-15 and PB-HCY-30). In brief, first, 20μl of EDTA plasma sample was mixed with 20μl reagent-R1 and 200μl reagent-R2. Next, the level of homocysteine was measured in a Randox fully automated analyzer. The test measures homocysteine content in the range of 2.5–80μmol/L.

### Preparation of bone marrow biopsy paraffin embedded blocks for immunohistochemical analysis

Paraffin blocks for immunohistochemical staining were prepared by first incubating the bone marrow biopsy specimens in 10% formalin for about 16h, followed by the decalcification using 10% EDTA treatment for 2–3 days [25]. Next, the EDTA was removed by washing the specimens thoroughly in running water, and subsequently processed using automated tissue processer (Thermo Scientific, Shandon Citadel-2000) as described below

1. Dehydration with 70% alcohol for 60 min followed by treatment with (a) 90% alcohol for 45 min (b) absolute alcohol for 45 min (2 times) and (c) absolute alcohol for 60 min.
2. The dehydrated blocks were treated with xylene for 60 min (3 times) followed by infiltration with paraffin wax for 30min, 60min and90 min.
3. At this point the blocks were ready for embedding using paraffin wax.
4. Once prepared, the paraffin blocks were sectioned using microtome—Leica RM 2255 to obtain 4μm thick sections. The sections were heat fixed on poly-L Lysine coated slides using a hot plate. The slides, thus prepared, were ready for immunohistochemical staining.

Immunohistochemical staining of tissue sections was carried out by the following procedure described in the product manual. First, the slides were deparaffinized with xylene (3 washes for 2–3 min) and hydrated using a graded alcohol series (2–3 min each). The hydrated slides were washed in running water and subjected to staining with p53 antibody (PathnSitu). In brief, first, the antigens were retrieved using EDTA buffer (10mM Tris Base, 0.37% EDTA, 0.5% Tween-20 and pH9.0) under steam pressure for 15 minutes followed by allowing the solution to cool for 10 minutes. Next, antigen retrieved slides were washed with TBS buffer (0.5M Tris Base, 0.9% NaCl, 0.5% Tween-20 and pH7.5–8.0) for 5 minutes and blocked with PolyExcel H_2_O_2_ for 5min. The peroxide blocked slides were washed once again with TBS buffer for 5 minutes and incubated with p53 primary antibody for 30 min at room temperature. The primary antibody was removed by washing with TBS buffer for 5 minutes and treated with PolyExcel Target Binder for 10min at room temperature. After washing with TBS buffer for 5 minutes the tissue sections were covered with PolyExcel PolyHRP conjugate for 10min at room temperature. At this point the slides washed once again with TBS buffer for 5minutes and a PolyExcel StunnDAB working solution was added. Next, the slides were allowed to stand for 5min at room temperature. These slides were washed with TBS buffer for 5minutes and stained with Hematoxylin (30 sec. at room temperature). Excess hematoxylin was removed by washing with tap-water and the slides dried by dehydrating through graded alcohols and xylenes. The dried slides were observed under Olympus BX-53 microscope, equipped with Olympus XC-10 camera. Two certified pathologists independently examined the slides and scored for the expression of p53. As suggested by supplier, the breast carcinoma tissue having wild type p53 was used as a control for positive staining while tissue from colon, not expressing p53, served as a negative control.

### Statistical analysis

All the results were subjected to statistical calculations usingStatistical Package for Social Science (SPSS). Linear association between two groups such as VB9 and VB12 levels with p53 expression among controls and cases was determined using Pearson's correlation of coefficient. The level of significance between two groups (such as Folic acid/Vitamin-B12/Homocysteine between control and cases) was determined using Kruskal-Wallis test.

## Results

### Megaloblastic anemia cases have abnormal blood cell counts and biochemical characteristics compared to non-megaloblastic anemia control subjects

The study subjects (n = 50; M = 31 and F = 19) exhibiting the characteristic megaloblastic anemia features were selected based on their clinical, biochemical and pathology reports. The control subjects (n = 50; M = 23 and F = 27) were the ones that were admitted in the hospital and undergoing bone marrow biopsy tests for medical problems other than megaloblastic anemia (Tables 1 and 2; and Fig 1a and 1b). Analysis and comparison of megaloblastic anemia and control subjects’ blood and bone marrow biopsy revealed a significantly (P<0.05) low RBC (Million cells/μL) (C = 2.42 ± 0.14 VS MBA = 1.92 ± 0.14), WBC (Cells/μL) (C = 5500 ± 670 VS MBA = 3380 ± 220) PLT (100000 Cells/μL) (C = 1.36 ± 0.19 VS MBA = 1.01 ± 0.13) counts. A significant increase in (a) MCV (fL/Red Cell) from 79.32 ± 1.67 to 107.44 ± 1.27; (b) RDW (%) from 19.94 ± 0.68 to 25.03 ± 0.65; and (c) ESR (mm/hr) from 59.28 ± 6.83 to 109.36 ± 5.52 was also observed in megaloblastic anemia cases. Furthermore, a statistically significant decrease in hemoglobin (g/dL) from 6.76 ± 0.38 to 5.44 ± 0.25 and reticulocyte count (%) from 2.43 ± 0.58 to 0.48 ± 0.07 was observed in megaloblastic anemia samples (Table 2). Analysis of bone marrow aspirate using Leishman’s stain showed a significant and distinct erythroid hyperplastic cells (Pink arrow) and open chromatin (Red arrow). Cells with more cytoplasm (Yellow arrow), giant sized nuclei (metamyelocytes, Black arrow) and band forms (neutrophils, Green arrow) and nuclear hyper-segmentation and abnormal maturation (megakaryocytes) were observed only in MBA cases but not in control subjects (Fig 1b).

**Table 1:** Demographics of Study Participants.

Demographic Parameter	Control	Case
Number of Participants	50	50
Age Mean Age	<40: 15	<40: 19
	>40: 35	>40: 31
	46.98	45.98
Gender	Male: 23, Female: 27	Male: 31, Female: 19

Controls participants are the subjects with no megaloblastic anemia, but have been referred by the clinicians for bone marrow biopsy examination for conditions other than megaloblastic anemia.

**Table 2:** Complete Blood Cells Count (CBC) Characteristics of Study Participants.

Characteristics	Controls	Cases	Normal Range
RBC (Million Cells / μL)	2.42 ± 0.14	1.92 ± 0.14	4.3 to 5.72 (Male) 3.9–5.03 (Female)
WBC (Cells/μL)	5500 ± 670	3380 ± 220	4000 to 11000 Cells/μL
PLT (Million/μL)	1.36 ± 0.19	1.01 ± 0.13	0.15–0.40 Million / μL
PCV (Hematocrit)(%)	19.45 ± 1.08	16.86 ± 0.78	35–47% (Female), 42–52% (Male)
MCV (fL/Red Cell)	79.32 ± 1.67	107.44 ± 1.27	80–96 fL/Red Cell
MCH*(pg/RBC)	27.31 ± 0.68	35.15 ± 0.56	26–33 pg/RBC.
MCHC (g/dL)	32.86 ± 0.41	33.51 ± 0.31	Normal value 28–33%
RDW (%)	19.94 ± 0.68	25.03 ± 0.65	Normal value 10.2–14.5%
ESR (mm/hr)	59.28 ± 6.83	109.36 ± 5.52	0–9 (Male), 10–20 (Female)
Hb (g/dL)	6.76 ± 0.38	5.44 ± 0.25	13–18 (Male), 12–16.5 (Female)
Retic (Reticulocyte count) (%)	2.43 ± 0.58	0.48 ± 0.07	0.2–2.0

Table shows Mean ± SD of complete blood count in controls (normocytic normochromic anemia, microcytic anemia and normocytic normochromic with microcytic anemia) and cases (megaloblastic anemia). Non-megaloblastic anemia control subjects showed characteristic low RBC, PCV and Hb. The megaloblastic anemia cases exhibited a drastic decrease in RBC, PCV, Hb and reticulocyte numbers with a significant increase in MCV and RDW.

* Calculated by multiplying the total amount of Hb with 10 and dividing with total number of RBCs

### Low Folic acid (Vitamin-B9) and Cyanocobalamin (Vitamin-B12), but high Homocysteine (HCys) content was observed in ~75% of Megaloblastic anemia subjects

**Fig 2:**
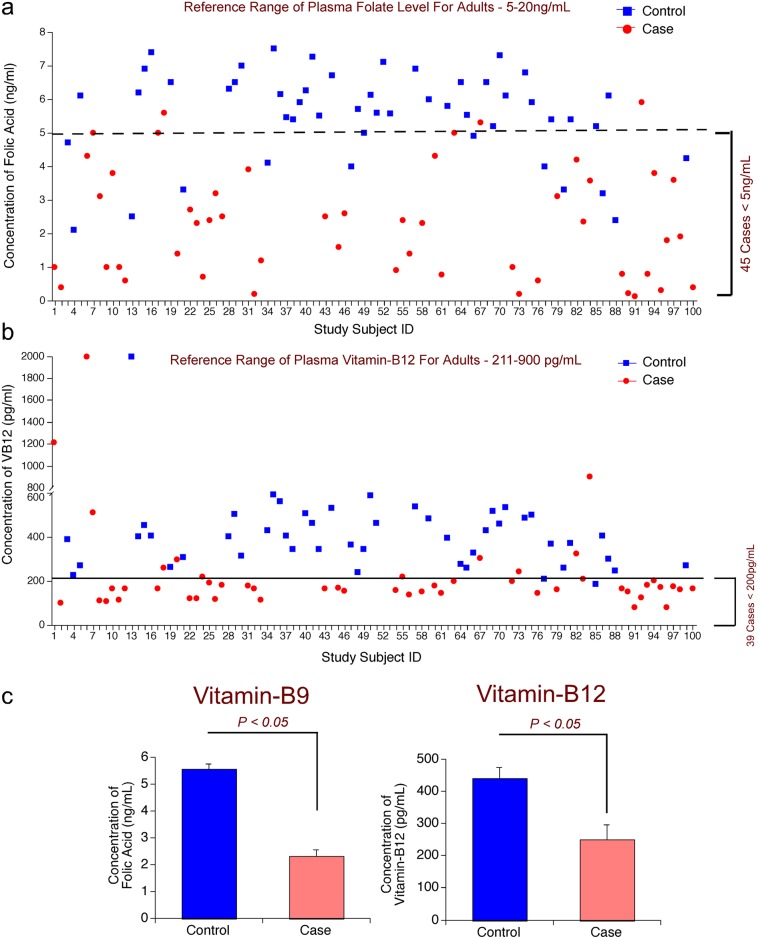
Patients Suffering From Megaloblastic Anemia Contain Low Vitamin-B9 And Vitamin-B12 In The Plasma. (a) Low vitamin-B9 (Folic Acid) levels were observed in 90% (45/50) cases: Compared to controls, 90% megaloblastic anemia cases contain low folate levels (<5.0ng/mL) in the plasma. However, only 26% (13/50) controls contain folate levels lower than normal (<5.0ng/mL). (b) Low vitamin-B12 levels were observed in 78% (39/50) cases: Compared to controls, 78% megaloblastic anemia cases contain low vitamin-B12 levels (<200pg/mL) in the plasma. All most all controls contain vitamin-B12 values higher than 200pg/mL. (c) Megaloblastic bone marrow cases contain at least 2 fold lower vitamin-B9 and vitamin-B12 levels compared to control subjects: Analysis of vitamin-B9 and vitamin-B12 in the plasma of control and megaloblastic anemia cases revealed at least a 2-fold decrease in the levels of these two important vitamins compared to control subjects.

Since megaloblastic anemic cases exhibited significant differences that are characteristic to this specific disease compared to control subjects, now we have determined whether such differences were also seen in the levels of vitamins, VB9 and VB12, which are known to play key roles in the development of RBCs; and the indicator of VB9 and VB12 deficiency ie., homocysteine [1]. The distribution of number of controls and cases with varied VB9 and VB12 is shown in (Table 3). Analysis of the levels of these vitamins and HCys showed a significant difference between control and megaloblastic anemic cases (Fig 2a, 2b and 2c). Whereas 12 control samples (out of 50) had less than the absolute VB9 deficiency level ie. <5.0ng/mL (normal range 5-20ng/mL, (Fig 2a, Table 4, and Table D in S1 File), 44 cases out of 50 megaloblastic anemia subjects had VB9 levels <5.0ng/mL (Fig 2a, Tables 5 and 6). Likewise analysis of the levels of VB12 showed a significantly low ie. <211pg/mL (normal range 211-900pg/mL) VB12 content in 40 cases (Fig 2b and Tables 6 and 7). However, only 2 controls showed cyanocobalamin level <200pg/mL (Fig 2b and Tables E and F in S1 File). Overall, the average folic acid and cyanocobalamin levels showed a significant 2-fold decrease in the megaloblastic anemia cases compared to controls (P < 0.05) (Fig 2c). Further analysis revealed that 74% megaloblastic anemia cases (37 out of 50 cases) had low levels of both VB9 and VB12 (Table 5); however, only 2% non-megaloblastic controls (1 out of 50; Subject #77; VB9—4ng/mL and VB12—209pg/ml) had both these vitamins in low levels (Table E in S1 File). Similarly, exceptional values were also observed in 3 megaloblastic anemia cases out of 50 total subjects (6%), which exhibited normalVB9 and VB12 levels (Table G in S1 File)

**Table 3:** Distribution of non-megaloblastic anemia control subjects and megaloblastic anemia cases based on VB9 and VB12 status.

VB9 and VB12 Status (VB9 (Normal range: 5-20ng/mL), VB12 (Normal Range: 211-911pg/mL)	Control (Non-megaloblastic anemia subjects (n = 50)	Case (Megaloblastic anemia subjects (n = 50)
VB9 and VB12 Low	01 (02%)	37 (74%)
VB9 Low, VB12 Normal	11 (22%)	07 (14%)
VB9 Normal, VB12 Low	01 (02%)	03 (06%)
VB9 and VB12 Normal	37 (74%)	03 (06%)

**Table 4:** Control non-megaloblastic anemia subjects with LOW VB9 (Normal range: 5.00ng/ml– 20ng/mL) but NORMAL VB12 (Normal range: 211.00–911.00pg/ml).

Sample No.	VB9 (ng/mL)	VB12 (pg/mL)	P53 expression (% of Total Cells)			
			Unstained	Low	Moderate	Heavy
3	4.70	389.00	22.45	25.66	37.29	14.57
4	2.10	227.00	25.14	21.55	28.35	24.95
13	2.50	2000.00	11.32	53.89	22.05	12.73
21	3.30	308.00	45.00	25.28	19.04	10.67
34	4.10	430.00	50.09	24.57	15.00	10.31
47	4.00	365.00	75.52	16.00	5.48	2.97
66	4.90	327.00	85.69	7.09	4.76	2.44
**77**	**4.00**	**209.00**	**46.80**	**30.91**	**15.35**	**6.92**
80	3.30	262.00	57.26	21.49	12.61	8.61
86	3.20	408.00	25.00	35.10	23.95	15.92
88	2.40	247.00	23.30	37.00	24.27	15.42
99	4.20	271.00	39.87	35.31	17.85	6.95
**N = 12**	Range = 2.10–4.90	Range = 209.00–2000.00	Range = 11.32–85.69	Range = 7.09–53.89	Range = 4.76–37.29	Range = 2.44–24.96
**Average**	3.55	453.58	42.28	27.82	18.83	11.03
**SD**	0.91	492.41	22.58	11.88	9.24	6.26
**SE**	0.27	142.31	6.64	3.49	2.71	1.84

**Table 5:** Megaloblastic anemia subjects with LOW VB9 and VB12 (Normal range: VB9–5.00–20.00ng/ml, VB12–211.00–911.00pg/ml).

Sample No.	VB9(ng/mL)	VB12(pg/mL)	P53 expression (% of Total Cells)			
			Unstained	Low	Moderate	Heavy
2	0.40	101.00	6.40	13.40	23.70	56.48
8	3.10	112.00	11.64	22.32	27.10	39.00
9	1.00	107.00	6.53	16.00	27.49	50.00
10	3.80	167.00	32.47	10.00	16.16	41.47
11	1.00	116.00	0.57	7.00	26.69	65.74
12	0.60	164.00	14.26	11.57	21.00	53.16
22	2.70	122.00	2.54	18.47	31.05	48.00
23	2.30	122.00	3.56	20.05	24.66	51.70
25	2.40	194.00	5.00	45.05	36.13	14.00
26	3.20	118.00	4.94	13.12	23.87	58.05
27	2.50	184.00	3.86	20.08	31.39	44.64
31	3.90	180.00	16.27	23.14	27.84	32.73
32	0.20	166.00	3.19	14.31	31.49	51.00
33	1.20	115.00	6.67	16.78	29.10	47.43
43	2.50	165.00	4.23	18.41	26.66	50.68
45	1.60	170.00	22.77	13.05	15.44	48.72
46	2.60	155.00	4.55	14.08	28.29	53.06
54	0.90	157.00	24.39	10.80	21.06	43.74
56	1.40	137.00	17.00	6.00	10.53	66.55
58	2.30	152.00	5.85	13.00	21.13	60.01
60	4.30	180.00	11.38	22.00	22.77	43.83
61	0.76	146.00	5.69	12.84	20.46	61.00
72	1.00	201.00	4.33	23.28	28.08	44.29
76	0.60	145.00	13.32	13.00	22.81	51.00
79	3.10	162.00	14.14	18.85	31.87	35.12
83	2.34	210.00	25.89	12.70	15.79	45.60
84	0.58	101.00	1.08	8.38	21.38	69.14
89	0.80	167.00	16.27	9.74	13.44	60.69
90	0.21	151.00	17.32	9.56	15.00	57.70
91	0.12	80.00	0.90	8.52	13.04	77.52
93	0.80	181.00	17.20	13.51	21.08	48.19
94	3.80	203.00	26.28	13.40	16.19	44.11
95	0.30	172.00	15.00	4.17	9.79	71.02
96	1.80	80.00	10.94	6.35	15.62	67.06
97	3.60	176.00	23.25	10.09	14.76	52.00
98	1.90	162.00	25.05	9.00	17.17	48.80
100	0.40	165.00	5.33	5.66	15.30	73.68
**N = 37**	Range = 0.2–4.3	Range = 80 210	Range = 0.57–32.47	Range = 4.17–45.05	Range = 9.79–31.87	Range = 14.0–77.52
**Aver**	1.78	150.97	11.62	14.26	22.03	52.07
**SD**	1.24	33.87	8.62	7.32	6.73	12.39
**SE**	0.20	5.57	1.41	1.20	1.10	2.03

**Table 6:** Megaloblastic Anemia subjects with LOW VB9 But NORMAL VB12 (Normal range: VB9–5.00–20.00ng/ml, VitB12–211.00–911.00pg/ml).

Sample No.	VB9 (ng/mL)	VitB12 (pg/mL)	P53 expression (% of Total Cells)			
			Unstained	Low	Moderate	Heavy
1	1.00	1215.00	24.67	22.63	23.19	29.49
6	4.30	2000.00	27.74	14.00	23.74	34.57
20	1.40	299.00	1.42	23.77	33.87	41.00
24	0.70	221.00	1.68	12.53	24.57	61.20
55	2.40	220.00	21.03	13.61	20.13	45.21
73	0.20	245.00	3.13	21.36	27.63	48.00
82	4.20	324.00	20.34	14.00	23.57	42.18
**N = 7**	Range = 0.20–4.30	Range = 220.00–2000.00	Range = 1.42–27.74	Range = 12.53–23.77	Range = 20.13–33.87	Range = 29.49–48.00
**Average**	2.03	646.28	14.28	17.41	25.24	43.09
**SD**	1.66	695.70	11.68	4.91	4.39	10.15
**SE**	0.62	263.52	4.42	1.86	1.66	3.84

**Table 7:** Megaloblastic anemia subjects with NORMAL VB9 BUT LOW VB12 (Normal range: VB9–5.00–20.00ng/mL, VitB12–211.00–911.00pg/mL).

Sample No.	VB9(ng/mL)	VitB12(pg/mL)	P53 expression (% of Total Cells)			
			Unstained	Low	Moderate	Heavy
17	5.00	166.00	3.81	26.00	27.20	43.00
63	5.00	201.00	10.58	20.13	25.59	43.68
92	5.90	124.00	16.63	25.22	25.00	33.09
**N = 3**	Range = 5.00–5.90	Range = 124.00–201.00	Range = 3.81–16.63	Range = 20.13–26.00	Range = 25.00–27.20	Range = 33.09–43.68
**Aver**	5.30	163.66	10.34	23.78	25.93	39.92
**SD**	0.51	38.55	6.41	3.18	1.13	5.92
**SE**	0.30	22.28	3.70	1.84	0.65	3.42

Note: Average (Aver), Standard deviation (SD), Standard error (SE).

**Fig 3:**
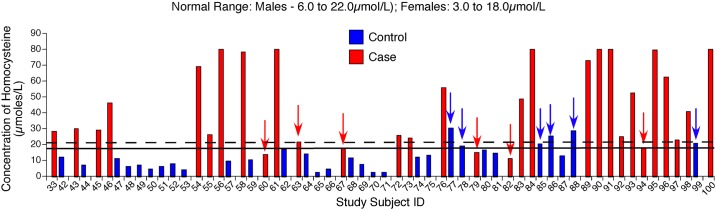
Patients Suffering From Megaloblastic Anemia Contain Very High Plasma Homocysteine (HCys) Compared to Non-Megaloblastic Anemia Subjects. Among the 30 megaloblastic anemia subjects 24 subjects (80%) had very high homocysteine levels in the plasma. However only 20% non-megaloblastic control subjects showed high plasma homocysteine. Therefore, very low VB9 and VB12, present in the megaloblastic anemia subjects, is responsible for elevated HCys in these subjects.

Since elevated homocysteine (HCys) is one of the key indicators of VB9 and VB12 deficiency, next, the level of HCys in30 non-megaloblastic controls and 30 megaloblastic anemia cases was measured. Only 30 samples, where sufficient quantity of plasma sample was available, were used for estimating HCys content using the protocol described in the materials and methods section. The data showed a high HCys (ie., >22μmol/L in males and > 18μmol/L in females) in 80% (24 out of 30 cases analyzed) MBA cases (Fig 3; Table H in S1 File). In contrast, only 20% non-MBA controls (6 out of 30 controls analyzed) had high HCys levels (Fig 3; Table I in S1 File). Over all an about 3.8 fold increase in homocysteine content from 11.97± 1.37μmol/L to 46.42 ±4.72 μmol/L was observed in megaloblastic anemia cases compared to non-megaloblastic control subjects (Tables H and I in S1 File).

### Elevated p53 expression was observed in megaloblastic anemia cases compared to control subjects

**Fig 4:**
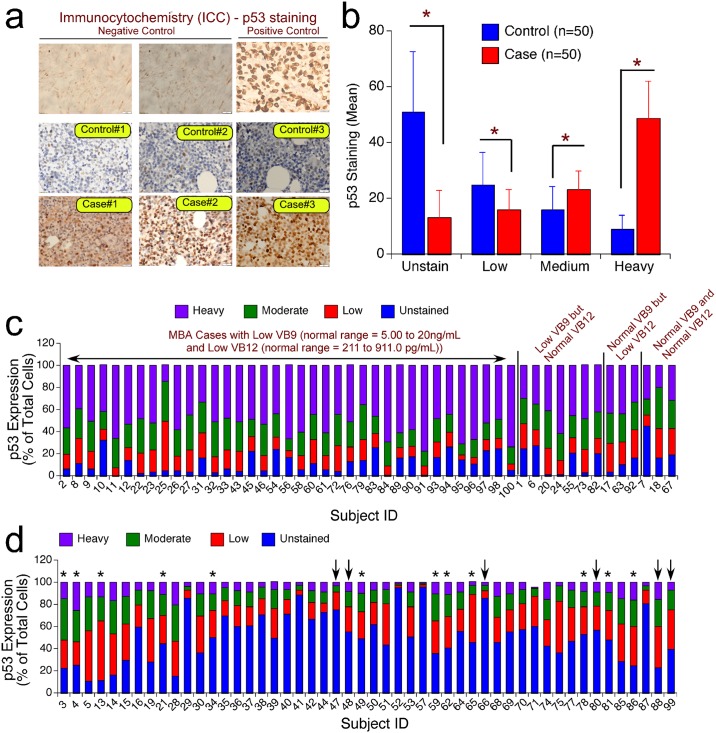
P53 Expression Is Elevated In The Megaloblastic Anemia. (a): Analysis of p53 expression in the bone marrow biopsies using immunocytochemistry: Bone marrow biopsy from control non-megaloblastic anemia, and cases with megaloblastic anemia were fixed and processed for immunohistochemical staining as described in materials and methods. The stained sections were observed under microscope and photographed using 40X magnification. The data showed more number of intensely expressed p53 protein in the megaloblastic anemia patients compared to control subjects. The breast carcinoma tissue with wild type p53 was used as positive control for p53 staining while the tissue from colon with no p53 expression served as negative control. (b) Quantitative assessment of p53 expression in megaloblastic anemia: Expression of p53 was quantified by counting the number of low, moderate (medium) and heavily stained cells (expressed p53 protein) and the results represented in terms of percentage total cells. The data showed a statistically significant ~5 fold increase in the heavily stained cells compared to controls. A similar percentage decrease was observed in the number of unstained cells, with more unstained cells observed in controls. (c) Relative distribution of p53 expressing cells with different degrees of staining in megaloblastic anemia subjects. MBA subjects with low VB9 and low VB12, low VB9 but normal VB12, normal VB9 but low VB12 and normal VB9 and normal VB12 were analyzed for the expression of p53 using IHC as described in materials and methods. The data showed a very high percentage p53 stained (heavy and moderate staining) cells in subjects deficient in VB9 and VB12 compared to subjects with normal VB9 and VB12 (P < 0.05) or subjects with having low VB9 or low VB12 (P < 0.05). (d) Relative distribution of p53 expressing cells with different degrees of staining in non-megaloblastic anemia control subjects. The graph shows the expression of p53 in different non-megaloblastic anemia subject categories—Normocytic Normochromic Anemia (NNA)– 31 individuals; Normocytic Normochromic and Microcytic Hypochromic Anemia (NNMA)–marked with * - 12 individuals; Microcytic Hypochromic Anemia (MHA)–marked with decrease—7 individuals. Further analysis showed the differences in p53 expression in subjects with low VB9 and low VB12 (only one individual ie., Subject #77), low VB9 but normal VB12 (Table 4), normal VB9 but low VB12 (Table e in S1 File, only one individual ie., Subject #85) and normal VB9 and normal VB12 (Refer Tables D and E in S1 File) were analyzed for the expression of p53 using IHC as described in materials and methods. The data showed a very high percentage (>75%) unstained and moderately stained (for p53) cells in 48 out of 50 subjects having at least normal VB12.

It is widely known that the megaloblastic red blood cells accumulate DNA aberrations and undergo apoptosis instead of progressing to mature RBCs [20]. However, it is not fully known how these megaloblastic red blood cells undergo apoptosis? In addition it is also not fully known which apoptotic protein is mediating the suicidal cell death of megaloblasts? And is there any link between the decreased folic acid and cyanocobalamin levels, as evidenced by increased homocysteine content, and the expression of apoptotic proteins such as p53? In order to address these gaps, first we have measured the expression of p53 in paraffin embedded blocks prepared using bone marrow biopsy and compared the expression levels with control non-megaloblastic samples. P53, also known as tumor protein53, is well known for its ability to induce apoptosis in cells with accumulated DNA mutations [16,26]. The immunohistochemical analysis of p53 protein showed a significant increase in the expression levels in the megaloblastic anemic cases compared to control paraffin embedded blocks (Fig 4a, 4b, 4c and 4d). Interestingly, the numbers of cells stained heavily for p53 expression are more in cases compared to controls, which had more number of unstained and mildly stained cells (Fig 4a, 4b, 4c and 4d). Further analysis determining the percentage unstained, low, moderate and heavily stained cells for p53 expression in control subjects having normal FB9 and FB12 (n = 37) showed the presence of more unstained (Average: 53.54% ± 3.43; range10.81% to 96.18%), and stained low (Average: 23.65% ± 1.87; range 2.12% to 46.46%) cells (Fig 4c,Table D in S1 File). But, in 37 cases containing low levels of VB9 and VB12 the percentage cells expressing high p53 protein are more (Average: 52.07% ± 2.03; range 14.00%– 77.52%) compared to unstained (Average: 11.62% ± 1.41; range 0.57% –32.47%) or stained low (Average: 14.26% ± 1.20 range 4.17% –45.05%) cell percentage (Fig 4d; Table 5).

Further analysis measuring the percentage (out of total number of cells including stained and unstained) of p53 stained cells with different degree of staining (light, moderate and heavy) in megaloblastic anemia cases with low VB9 (<5.0ng/mL) and VB12 (<211pg/mL) (n = 37) showed a high abundance of heavily stained (Average: 52.08 ± 2.03; ranging from 14% to 77.52%) and moderately stained (Average: 22.03 ± 1.10; ranging from 9.79% to 31.87%) cells compared to low- and unstained cells, which are in the range from 4.17% to 45.05% (Average: 14.26 ± 1.20) and 0.57% to 32.47% (Average: 11.62 ± 1.41), respectively (Table 5). In essence, the total percentage of p53 stained cells ranged from 67.53% to 99.43% in megaloblastic cases where both VB9 and VB12 are significantly lower than normal cells (Table 5). However, the number of cells expressing p53 is much lower in majority of non-megaloblastic controls as 48 out of 50 control subjects contain one of these vitamins (VB 9 or VB12) in normal levels (Table D in S1 File (for normal VB9), and Table F in S1 File (for normal VB12). For example, 48 subjects having normal VB12 showed high percentage of unstained cells (Average: 51.38 ± 3.16; range 10.81 to 96.18) but less percentage of p53 stained cells (Table F in S1 File). Among the p53 stained cells, the percentage cells stained low is more compared to (Average: 24.32 ± 1.69; 2.12 to 46.46), moderate (Average: 15.59 ± 1.20; range 1.13 to 33.00), and heavily (Average: 8.59 ± 0.73; range 0.55 to 20.00) stained cells (Table F in S1 File). Similarly, the control subjects with normal VB9 also expressed a high percentage unstained cells (Average: 53.53 ± 3.43; range 10.81–96.18) compared to p53 expressing cells with low (Average: 23.64 ± 1.87); moderate (Average: 14.75 ± 1.26) and heavy (Average: 7.93 ± 0.72) stained cells (Table D in S1 File). Interestingly, in controls subjects with low VB9 (n = 12) or VB12 (n = 2) the percentage p53 positive cells was slightly higher compared to subjects with normal VB9 and VB12 levels (Table 4 and Table E in S1 File). Only one subject (Subject ID #77) had low VB9 and VB12 among all non-megaloblastic anemia controls (Table F in S1 File). The expression of p53 in this subject showed 46.8% unstained cells and 30.91 low stained cells. Only 15.35% and 6.92% moderately and heavily stained p53 cells were observed in this subject (Table E in S1 File).

## Discussion

Megaloblastic anemia (MBA), caused primarily by low vitamin B12 (VB12, Cyanocobalamin) and / or decreased Vitamin B9 (VB9, Folate), is characterized by abnormally large erythrocytes (megaloblasts) and is responsible for many neuro disorders [27,28,29]. Megaloblast cells are characterized by the presence of abnormal nuclear material, which is due to the imbalance between nuclear maturation and cytoplasmic contents. Furthermore, megaloblasts exhibit impaired DNA synthesis and S-phase cell cycle arrest [28].

Even though the Office of Rare Disorders of NIH, USA, lists MBA as a “Rare Disorder” the number of individuals suffering from this disease is high in developing countries [30]. Therefore, necessary preventive and treatment measures need to be taken to control this disease. Better understanding of this disease at molecular level helps to develop more effective treatment strategies. Hence, in this present study, first, we have measured the levels of two important regulators of MBA, viz, Folic acid (Vitamin-B9) and Cyanocobalamin (Vitamin-B12) along with homocysteine, an indicator of VB9 and VB12 deficiency, in the plasma of control subjects who are suffering from anemia other than MBA and referred by the doctor for bone marrow examination, and patients suffering from MBA. As predicted a statistically significant low vitamin-B9 and Vitamin-B12 were observed in ~75% of MBA cases compared to non-megaloblastic controls indicating that MBA is primarily due to the deficiency of VB9 and VB12. Homocysteine level is a key sulfur containing amino acid produced from methionine and is an indicator of low plasma VB9 and VB12. Since plasma homocysteine gets remethylated in the presence of VB9 and VB12 a significant deficiency in the levels of these two vitamins cause an accumulation of homocysteine in the plasma. Elevated plasma homocysteine is one of the important risk factors for various pathological conditions that include cardiovascular diseases, stroke and neurodegeneration [31]. Therefore, diets supplemented with VB9 and VB12 are recommended for individuals with elevated homocysteine content. In this study we have shown that 80% control non-megaloblastic subjects have normal levels of homocysteine. However, the concentration of homocysteine in the plasma is much higher in cases 80% cases.

Due to low vitamin-B12 and B9 levels the hematopoietic progenitor cells (a) lose their ability to repair DNA damage; (b) mis-incorporate uracil in place of thymidylate; and (c) arrest cells in the G1/S phase of the cell cycle [32]. Ultimately the cells undergo apoptosis by upregulating several proteins involved in suicidal death mechanisms. A prior study has demonstrated apoptosis induction in megaloblastic anemia cells by a p53-independent mechanism by demonstrating that p53-null mice fed with folate deficient diet accumulated megaloblastic cells in S-phase [20]. In addition, it has also been showed by these investigators that the unusual megaloblasts undergo apoptosis by a p53 independent mechanism [20]. In contrary, a recent study showed a p53-dependent mechanism in the development of macrocytic anemia in a mouse model representing the 5q-syndrome [33]. Many other studies using carcinoma cells have also demonstrated the induction of p53 when the cells were starved of folate [34]. Therefore, to address these contrary issues about the p53 expression in megaloblastic anemic patients, bone marrow aspirates from 50 cases with confirmed megaloblastic anemia were collected and paraffin embedded blocks analyzed using immunohistochemistry for the expression of p53. A significant increase in the moderately and heavily stained cells was observed in bone marrow biopsies collected from MBA patients indicating that low vitamin-B12 and vitamin-B9 elevated the p53 expression as a compensatory mechanism initially to repair the damaged DNA (due to mis-incorporation of Uracil in place of Thymidine) as well as to eliminate the cells if irreparable by triggering the apoptosis. P53 is known as a guardian of genome and known to protect cellular DNA from the damage induced by various insults [26]. If the damage is irreparable, then the p53 protein prevent the entry of cells in to S-phase by trans-activating the expression of p21^WAF1^ or by increasing the levels of GADD45 and 14-3-3σ, which control the G2-M transition [35,36]. Any additional damages to these cells trigger suicidal death mechanisms by up regulating the expression of Caspase-9/Apaf-1 pathway proteins or by triggering the release of cytochrome-c from mitochondria as well as by promoting Bax, Noxa, PUMA etc [37,38,39,40]. Therefore, p53 is a master protector of nuclear DNA and its expression gets elevated if any damage occurs to the cellular DNA as observed in the present study.

## Conclusion

In conclusion the results from our study demonstrate that p53 is heavily expressed in the megaloblastic cells of patients suffering from megaloblastic anemia. Elevated p53 expression in MBA is predicted to be due to very low levels of vitamin-B12 and vitamin-B9 in megaloblastic anemia cases compared to controls that are suffering from anemia other than MBA. These results also suggest that measuring the expression of p53 in the anemic patients helps in the diagnosis of MBA as the p53 expression is specifically up regulated in the MBA samples but not in the other anemic samples.

## Supporting Information

S1 File Tables A-I. A: VB9, VB12 and Homocysteine levels and p53 expression pattern in Normocytic Normochromic Anemia (NNA) control subjects (Normal range Males: 6–22μmol/L; Females: 3–18 μmol/L). B: VB9, VB12, Homocysteine levels and p53 expression pattern in Normocytic Normochromic and Microcytic Hypochromic Anemia (NNMA) control subjects (Normal range Males: 6–22μmol/L; Females: 3–18 μmol/L). C: VB9, VB12 and Homocysteine levels and p53 expression pattern in Microcytic Hypochromic Anemia (MHA) control subjects (Normal range Males: 6–22μmol/L; Females: 3–18 μmol/L). D: Control non-megaloblastic anemia subjects with normal VB9 levels (Normal range ≥5.00–20.00ng/ml). E: Control non-megaloblastic anemia subjects with LOW VB12 (Normal range: 211.00–911.00pg/ml). F: Control non-megaloblastic anemia subjects with normal VB12 (Normal range: 211.00–911.00pg/mL). G: Megaloblastic anemia subjects with normal VB9 and VB12 (Normal range: VB9–5.00–20.00ng/mL, VitB12–211.00–911.00pg/mL). H: Homocysteine and p53 expression pattern in Megaloblastic anemia cases (Normal range Males: 6–22μmol/L; Females: 3–18 μmol/L). I: Homocysteine and p53 expression pattern in non-megaloblastic anemia subjects (Normal range Males: 6–22μmol/L; Female: 3–18 μmol/L).
(DOCX)
