# Supplementary material for: Comparative Assessment of Vitamin-B12, Folic Acid and Homocysteine Levels in Relation to p53 Expression in Megaloblastic Anemia
Source: PLoS One. 2016 Oct 25;11(10):e0164559. doi: 10.1371/journal.pone.0164559 (PMC5079580; doi:10.1371/journal.pone.0164559)
Supplement: S1 File — A: VB9, VB12 and Homocysteine levels and p53 expression pattern in Normocytic Normochromic Anemia (NNA) control subjects (Normal range Males: 6–22μmol/L; Females: 3–18 μmol/L). B: VB9, VB12, Homocysteine levels and p53 expression pattern in Normocytic Normochromic and Microcytic Hypochromic Anemia (NNMA) control subjects (Normal range Males: 6–22μmol/L; Females: 3–18 μmol/L). C: VB9, VB12 and Homocysteine levels and p53 expression pattern in Microcytic Hypochromic Anemia (MHA) control subjects (Normal range Males: 6–22μmol/L; Females: 3–18 μmol/L). D: Control non-megaloblastic anemia subjects with normal VB9 levels (Normal range ≥5.00–20.00ng/ml). E: Control non-megaloblastic anemia subjects with LOW VB12 (Normal range: 211.00–911.00pg/ml). F: Control non-megaloblastic anemia subjects with normal VB12 (Normal range: 211.00–911.00pg/mL). G: Megaloblastic anemia subjects with normal VB9 and VB12 (Normal range: VB9–5.00–20.00ng/mL, VitB12–211.00–911.00pg/mL). H: Homocysteine and p53 expression pattern in Megaloblastic anemia cases (Normal range Males: 6–22μmol/L; Females: 3–18 μmol/L). I: Homocysteine and p53 expression pattern in non-megaloblastic anemia subjects (Normal range Males: 6–22μmol/L; Female: 3–18 μmol/L). (DOCX) [file pone.0164559.s001.docx]

**Table A: VB9, VB12 and Homocysteine levels and p53 expression pattern in Normocytic Normochromic Anemia (NNA) control subjects (Normal range Males: 6 – 22µmol/L; Females: 3-18 µmol/L)**

| **Normocytic Normochromic Anemia Controls** | | | | | **P53 expression (% of Total Cells)** | | | | |
| --- | --- | --- | --- | --- | --- | --- | --- | --- | --- |
| **Sample No.** | **VB9 (ng/mL)** | **VitB12 (pg/mL)** | | **HCys (µmol/L)** | **Unstained** | **Low** | **Medium** | | **Heavy** |
| 5 | 6.10 | 271.00 | | ND | 10.81 | 45.58 | 30.38 | | 13.21 |
| 14 | 6.20 | 405.00 | | ND | 16.63 | 37.32 | 29.80 | | 16.23 |
| 15 | 6.90 | 454.00 | | ND | 29.57 | 33.26 | 24.90 | | 12.25 |
| 16 | 7.40 | 407.00 | | ND | 59.77 | 19.79 | 13.61 | | 6.80 |
| 19 | 6.50 | 263.00 | | ND | 28.32 | 39.45 | 25.05 | | 7.17 |
| 28 | 6.30 | 405.00 | | ND | 15.50 | 31.29 | 33.00 | | 20.00 |
| 29 | 6.50 | 506.00 | | ND | 85.85 | 7.24 | 3.57 | | 3.31 |
| 30 | 7.00 | 315.00 | | ND | 36.25 | 33.77 | 19.61 | | 10.35 |
| 35 | 7.50 | 594.00 | | ND | 69.69 | 13.78 | 11.74 | | 4.77 |
| 36 | 6.15 | 565.00 | | ND | 60.22 | 19.12 | 13.30 | | 7.34 |
| 37 | 5.45 | 407.00 | | ND | 61.08 | 17.05 | 13.54 | | 8.32 |
| 38 | 5.39 | 347.00 | | ND | 70.86 | 14.75 | 10.17 | | 4.19 |
| 39 | 5.90 | 604.00 | | ND | 50.00 | 26.47 | 17.03 | | 6.57 |
| 40 | 6.25 | 510.00 | | ND | 71.40 | 13.14 | 9.41 | | 6.03 |
| 41 | 7.25 | 465.00 | | ND | 88.91 | 4.34 | 4.13 | | 2.60 |
| 42 | 5.50 | 345.00 | | 11.80 | 66.87 | 15.19 | 10.73 | | 7.19 |
| 44 | 6.70 | 534.00 | | 7.00 | 72.94 | 8.75 | 9.85 | | 8.45 |
| 50 | 6.12 | 590.00 | 4.40 | | 61.88 | 20.09 | 11.00 | 7.00 | |
| 51 | 5.60 | 465.00 | 6.00 | | 43.47 | 37.43 | 12.56 | 6.52 | |
| 52 | 7.10 | 610.00 | 7.60 | | 95.28 | 2.46 | 1.21 | 1.03 | |
| 53 | 5.56 | 680.00 | 3.90 | | 50.67 | 27.28 | 13.55 | 8.47 | |
| 57 | 6.90 | 539.00 | 9.50 | | 96.18 | 2.12 | 1.13 | 0.56 | |
| 59 | 6.00 | 484.00 | 10.30 | | 36.07 | 29.42 | 24.44 | 10.05 | |
| 64 | 6.50 | 278.00 | 13.90 | | 55.84 | 20.00 | 15.37 | 8.85 | |
| 68 | 6.50 | 430.00 | 11.70 | | 46.02 | 22.52 | 19.00 | 12.47 | |
| 69 | 5.20 | 520.00 | 7.40 | | 55.34 | 19.87 | 14.79 | 10.00 | |
| 70 | 7.30 | 462.00 | 2.50 | | 57.53 | 23.61 | 12.43 | 6.41 | |
| 71 | 6.10 | 535.00 | 2.50 | | 60.23 | 27.15 | 7.66 | 0.55 | |
| 74 | 6.80 | 490.00 | 12.10 | | 42.48 | 23.79 | 18.68 | 15.03 | |
| 75 | 5.90 | 502.00 | 13.00 | | 36.61 | 46.46 | 13.70 | 3.21 | |
| 87 | 6.10 | 300.00 | 12.70 | | 80.91 | 12.10 | 4.10 | 2.87 | |
| **N=31** | Range 5.39 – 7.50 | Range 263.00 – 680.00 | Range 2.50 – 13.90 | | Range 10.81 - 96.18 | Range 2.12 – 46.46 | Range 1.13 – 33.00 | Range 0.55 – 16.23 | |

ND: Note determined

**Table B: VB9, VB12, Homocysteine levels and p53 expression pattern in Normocytic Normochromic and Microcytic Hypochromic Anemia (NNMA) control subjects (Normal range Males: 6 – 22µmol/L; Females: 3-18 µmol/L)**

| **Normocytic Normochromic and Microcytic Hypochromic Anemia (NNMA)** | | | | **P53 expression (% of Total Cells)** | | | |
| --- | --- | --- | --- | --- | --- | --- | --- |
| **Sample No.** | **VB9**  **(ng/mL)** | **VitB12**  **(pg/mL)** | **HCys**  **(µmol/L)** | **Unstained** | **Low** | **Moderate** | **Heavy** |
| 3 | 4.70 | 389.00 | ND | 22.45 | 25.66 | 37.29 | 14.57 |
| 4 | 2.10 | 227.00 | ND | 25.14 | 21.55 | 28.35 | 24.95 |
| 13 | 2.50 | 2000.00 | ND | 11.32 | 53.89 | 22.05 | 12.73 |
| 21 | 3.30 | 308.00 | ND | 45.00 | 25.28 | 19.04 | 10.67 |
| 34 | 4.10 | 430.00 | ND | 50.09 | 24.57 | 15.00 | 10.31 |
| 49 | 5.00 | 345.00 | 7.10 | 49.14 | 24.41 | 16.64 | 9.79 |
| 62 | 5.80 | 398.00 | 17.10 | 41.00 | 28.14 | 20.11 | 10.77 |
| 65 | 5.50 | 262.00 | 2.50 | 46.06 | 43.00 | 8.36 | 2.65 |
| 78 | 5.40 | 370.00 | 18.90 | 53.18 | 24.68 | 14.02 | 8.11 |
| 81 | 5.40 | 372.00 | 14.60 | 47.87 | 27.00 | 15.79 | 9.32 |
| 85 | 5.20 | 187.00 | 20.20 | 28.71 | 33.86 | 22.69 | 14.70 |
| 86 | 3.20 | 408.00 | 25.10 | 25.00 | 35.10 | 23.95 | 15.92 |
| **N = 12** | Range = 2.10 – 6.00 | Range =  187.00 – 2000.00 | Range = 2.50 – 25.10 | Range = 11.32 – 53.18 | Range = 21.55 - 53.89 | Range = 8.36 – 37.29 | Range = 2.65 – 24.95 |

ND: Not determined

**Table C: VB9, VB12 and Homocysteine levels and p53 expression pattern in Microcytic Hypochromic Anemia (MHA) control subjects (Normal range Males: 6 – 22µmol/L; Females: 3-18 µmol/L)**

| **Microcytic Hypochromic Anemia (MHA)** | | | | | **P53 expression (% of Total Cells)** | | | |
| --- | --- | --- | --- | --- | --- | --- | --- | --- |
| **Sample No.** | **VB9**  **(ng/mL)** | **VitB12**  **(pg/mL)** | **HCys**  **(µmol/L)** | | **Unstained** | **Low** | **Moderate** | **Heavy** |
| 47 | 4.00 | 365.00 | 11.00 | | 75.52 | 16.00 | 5.48 | 2.97 |
| 48 | 5.70 | 240.00 | 6.00 | | 55.30 | 23.00 | 13.46 | 8.30 |
| 66 | 4.90 | 327.00 | 4.50 | | 85.69 | 7.09 | 4.76 | 2.44 |
| 77 | 4.00 | 209.00 | 30.30 | | 46.80 | 30.91 | 15.35 | 6.92 |
| 80 | 3.30 | 262.00 | 16.60 | | 57.26 | 21.49 | 12.61 | 8.61 |
| 88 | 2.40 | 247.00 | | 28.40 | 23.30 | 37.00 | 24.27 | 15.42 |
| 99 | 4.20 | 271.00 | | 20.70 | 39.87 | 35.31 | 17.85 | 6.95 |
| N = 7 | Range = 2.40 – 5.70 | Range = 209 – 365.00 | | Range = 4.50 – 30.30 | Range = 23.30 – 85.69 | Range = 7.09 – 37.00 | Range = 4.76 – 24.27 | Range = 2.44 – 15.42 |

**Table D: Control non-megaloblastic anemia subjects with normal VB9 levels (Normal range ≥5.00 – 20.00ng/ml):**

| **Sample No.** | **VB9**  **(ng/mL)** | **VB12 (pg/mL)** | **P53 expression (% of Total Cells)** | | | |
| --- | --- | --- | --- | --- | --- | --- |
|  | | | **Unstained** | **Low** | **Moderate** | **Heavy** |
| 5 | 6.10 | 271.00 | 10.81 | 45.58 | 30.38 | 13.21 |
| 14 | 6.20 | 405.00 | 16.63 | 37.32 | 29.80 | 16.23 |
| 15 | 6.90 | 454.00 | 29.57 | 33.26 | 24.90 | 12.25 |
| 16 | 7.40 | 407.00 | 59.77 | 19.79 | 13.61 | 6.80 |
| 19 | 6.50 | 263.00 | 28.32 | 39.45 | 25.05 | 7.17 |
| 28 | 6.30 | 405.00 | 15.50 | 31.29 | 33.00 | 20.00 |
| 29 | 6.50 | 506.00 | 85.85 | 7.24 | 3.57 | 3.31 |
| 30 | 7.00 | 315.00 | 36.25 | 33.77 | 19.61 | 10.35 |
| 35 | 7.50 | 594.00 | 69.69 | 13.78 | 11.74 | 4.77 |
| 36 | 6.10 | 565.00 | 60.22 | 19.12 | 13.30 | 7.34 |
| 37 | 5.40 | 407.00 | 61.08 | 17.05 | 13.54 | 8.32 |
| 38 | 5.40 | 347.00 | 70.86 | 14.75 | 10.17 | 4.19 |
| 39 | 5.90 | 604.00 | 50.00 | 26.47 | 17.03 | 6.57 |
| 40 | 6.20 | 510.00 | 71.40 | 13.14 | 9.41 | 6.03 |
| 41 | 7.20 | 465.00 | 88.91 | 4.34 | 4.13 | 2.60 |
| 42 | 5.50 | 345.00 | 66.87 | 15.19 | 10.73 | 7.19 |
| 44 | 6.70 | 534.00 | 72.94 | 8.75 | 9.85 | 8.45 |
| 48 | 5.70 | 240.00 | 55.30 | 23.00 | 13.46 | 8.30 |
| 49. | 5.00 | 345.00 | 49.14 | 24.41 | 16.64 | 9.79 |
| 50 | 6.10 | 590.00 | 61.88 | 20.09 | 11.00 | 7.00 |
| 51 | 5.60 | 465.00 | 43.47 | 37.43 | 12.56 | 6.52 |
| 52 | 7.10 | 610.00 | 95.28 | 2.46 | 1.21 | 1.03 |
| 53 | 5.50 | 680.00 | 50.67 | 27.28 | 13.55 | 8.47 |
| 57 | 6.90 | 539.00 | 96.18 | 2.12 | 1.13 | 0.56 |
| 59 | 6.00 | 484.00 | 36.07 | 29.42 | 24.44 | 10.05 |
| 62 | 5.80 | 398.00 | 41.00 | 28.14 | 20.11 | 10.77 |
| 64 | 6.50 | 278.00 | 55.84 | 20.00 | 15.37 | 8.85 |
| 65 | 5.50 | 262.00 | 46.06 | 43.00 | 8.36 | 2.65 |
| 68 | 6.50 | 430.00 | 46.02 | 22.52 | 19.00 | 12.47 |
| 69. | 5.20 | 520.00 | 55.34 | 19.87 | 14.79 | 10.00 |
| 70 | 7.30 | 462.00 | 57.53 | 23.61 | 12.43 | 6.41 |
| 71 | 6.10 | 535.00 | 60.23 | 27.15 | 7.66 | 0.55 |
| 74 | 6.80 | 490.00 | 42.48 | 23.79 | 18.68 | 15.03 |
| 75 | 5.90 | 502.00 | 36.61 | 46.46 | 13.70 | 3.21 |
| 78 | 5.40 | 370.00 | 53.18 | 24.68 | 14.02 | 8.11 |
| 81 | 5.40 | 372.00 | 47.87 | 27.00 | 15.79 | 9.32 |
| 85. | 5.20 | 187.00 | 28.71 | 33.86 | 22.69 | 14.70 |
| 87 | 6.10 | 300.00 | 80.91 | 12.10 | 4.10 | 2.87 |
| **N=38** | Range =  5.00 – 7.40 | Range = 187.00 – 680.00 | Range = 10.81 to 96.18 | Range = 2.12 to 46.46 | Range = 1.13 to 33.00 | Range = 0.55 to 20.00 |
| **Average** | 6.17 |  | 53.54 | 23.65 | 14.75 | 7.93 |
| **SD** | 0.68 |  | 20.95 | 11.44 | 7.728 | 4.43 |
| **Errors** | 0.11 |  | 3.43 | 1.87 | 1.26 | 0.72 |

**Table E: Control non-megaloblastic anemia subjects with LOW VB12 (Normal range: 211.00-911.00pg/ml):**

| **Sample No.** | **VB9 (ng/mL)** | **VB12**  **(pg/mL)** | **P53 expression (% of Total Cells)** | | | |
| --- | --- | --- | --- | --- | --- | --- |
|  | |  | **Unstained** | **Low** | **Moderate** | **Heavy** |
| **77** | **4.00** | **209.00** | **46.80** | **30.91** | **15.35** | **6.92** |
| 85 | 5.20 | 187.00 | 28.71 | 33.86 | 22.69 | 14.70 |
| **N=2** | Range = 4.00 to 5.20 | Range = 187 - 209 | Range = 28.71 to 46.80 | Range = 30.91 to 33.86 | Range = 15.35 to 22.69 | Range = 6.92 to 14.70 |
| **Average** | 4.60 | 198.00 | 37.75 | 32.38 | 19.02 | 10.81 |

**Table F: Control non-megaloblastic anemia subjects with normal VB12 (Normal range: 211.00-911.00pg/mL):**

| **Sample No.** | **VB9 (ng/mL)** | **VB12**  **(pg/mL)** | **P53 expression (% of Total Cells)** | | | |
| --- | --- | --- | --- | --- | --- | --- |
|  | | | **Unstained** | **Low** | **Moderate** | **Heavy** |
| 3 | 4.70 | 389.00 | 22.45 | 25.66 | 37.29 | 14.57 |
| 4 | 2.10 | 227.00 | 25.14 | 21.55 | 28.35 | 24.95 |
| 5 | 6.10 | 271.00 | 10.81 | 45.58 | 30.38 | 13.21 |
| 13 | 2.50 | **2000.00** | 11.32 | 53.89 | 22.05 | 12.73 |
| 14 | 6.20 | 405.00 | 16.63 | 37.32 | 29.80 | 16.23 |
| 15 | 6.90 | 454.00 | 29.57 | 33.26 | 24.90 | 12.25 |
| 16 | 7.40 | 407.00 | 59.77 | 19.79 | 13.61 | 6.80 |
| 19 | 6.50 | 263.00 | 28.32 | 39.45 | 25.05 | 7.17 |
| 21 | 3.30 | 308.00 | 45.00 | 25.28 | 19.04 | 10.67 |
| 28 | 6.30 | 405.00 | 15.50 | 31.29 | 33.11 | 20.10 |
| 29 | 6.50 | 506.00 | 85.85 | 7.24 | 3.57 | 3.31 |
| 30 | 7.00 | 315.00 | 36.25 | 33.77 | 19.61 | 10.35 |
| 34 | 4.10 | 430.00 | 50.09 | 24.57 | 15.00 | 10.31 |
| 35 | 7.50 | 594.00 | 69.69 | 13.78 | 11.74 | 4.77 |
| 36 | 6.10 | 565.00 | 60.22 | 19.12 | 13.30 | 7.34 |
| 37 | 5.40 | 407.00 | 61.08 | 17.05 | 13.54 | 8.32 |
| 38 | 5.40 | 347.00 | 70.86 | 14.75 | 10.17 | 4.19 |
| 39 | 5.90 | 604.00 | 50.00 | 26.47 | 17.03 | 6.57 |
| 40 | 6.20 | 510.00 | 71.40 | 13.14 | 9.41 | 6.03 |
| 41 | 7.20 | 465.00 | 88.91 | 4.34 | 4.13 | 2.60 |
| 42 | 5.50 | 345.00 | 66.87 | 15.19 | 10.73 | 7.19 |
| 44 | 6.70 | 534.00 | 72.94 | 8.75 | 9.85 | 8.45 |
| 47 | 4.00 | 365.00 | 75.52 | 16.00 | 5.48 | 2.97 |
| 48 | 5.70 | 240.00 | 55.30 | 23.00 | 13.46 | 8.30 |
| 49 | 5.00 | 345.00 | 49.14 | 24.41 | 16.64 | 9.79 |
| 50 | 6.10 | 590.00 | 61.88 | 20.09 | 11.00 | 7.00 |
| 51 | 5.60 | 465.00 | 43.47 | 37.43 | 12.56 | 6.52 |
| 52 | 7.10 | 610.00 | 95.28 | 2.46 | 1.21 | 1.03 |
| 53 | 5.50 | 680.00 | 50.67 | 27.28 | 13.55 | 8.47 |
| 57 | 6.90 | 539.00 | 96.18 | 2.12 | 1.13 | 0.56 |
| 59 | 6.00 | 484.00 | 36.07 | 29.42 | 24.44 | 10.05 |
| 62 | 5.80 | 398.00 | 41.00 | 28.14 | 20.11 | 10.77 |
| 64 | 6.50 | 278.00 | 55.84 | 20.00 | 15.37 | 8.85 |
| 65 | 5.50 | 262.00 | 46.06 | 43.00 | 8.36 | 2.65 |
| 66 | 4.90 | 327.00 | 85.69 | 7.09 | 4.76 | 2.44 |
| 68 | 6.50 | 430.00 | 46.02 | 22.52 | 19.00 | 12.47 |
| 69 | 5.20 | 520.00 | 55.34 | 19.87 | 14.79 | 10.00 |
| 70 | 7.30 | 462.00 | 57.53 | 23.61 | 12.43 | 6.41 |
| 71 | 6.10 | 535.00 | 60.23 | 27.15 | 7.66 | 0.55 |
| 74 | 6.80 | 490.00 | 42.48 | 23.79 | 18.68 | 15.03 |
| 75 | 5.90 | 502.00 | 36.61 | 46.46 | 13.70 | 3.21 |
| 78 | 5.40 | 370.00 | 53.18 | 24.68 | 14.02 | 8.11 |
| 80 | 3.30 | 262.00 | 57.26 | 21.49 | 12.61 | 8.61 |
| 81 | 5.40 | 372.00 | 47.87 | 27.00 | 15.79 | 9.32 |
| 86 | 3.20 | 408.00 | 25.00 | 35.10 | 23.95 | 15.92 |
| 87 | 6.10 | 300.00 | 80.91 | 12.10 | 4.10 | 2.87 |
| 88 | 2.40 | 247.00 | 23.30 | 37.00 | 24.27 | 15.42 |
| 99 | 4.20 | 271.00 | 39.87 | 35.31 | 17.85 | 6.95 |
| **N = 48** | Range = 2.10 to 7.50 | Range = 227.00 to 2000.00 | Range = 10.81 to 96.18 | Range = 2.12 to 46.46 | Range = 1.13 to 33.00 | Range = 0.55 to 20.00 |
| **Average** |  | 447.98 | 51.38 | 24.33 | 15.59 | 8.59 |
| **SD** |  | 255.88 | 21.87 | 11.70 | 8.32 | 5.07 |
| **Errors** |  | 36.98 | 3.16 | 1.69 | 1.20 | 0.73 |

**Table G: Megaloblastic anemia subjects with normal VB9 and VB12 (Normal range: VB9 - 5.00 – 20.00ng/mL, VitB12 – 211.00-911.00pg/mL):**

| **Sample No.** | **VB9**  **(ng/mL)** | **VitB12**  **(pg/mL)** | **P53 expression (% of Total Cells)** | | | |
| --- | --- | --- | --- | --- | --- | --- |
|  | | | **Unstained** | **Low** | **Moderate** | **Heavy** |
| 7 | 5.00 | 514.00 | 45.11 | 10.00 | 14.45 | 30.38 |
| 18 | 5.60 | 262.00 | 16.37 | 27.00 | 36.93 | 19.68 |
| 67 | 5.30 | 305.00 | 19.39 | 23.91 | 25.58 | 31.10 |
| **N=3** | Range = 5.00 - 5.60 | Range = 262.00-514.00 | Range =  16.37- 45.11 | Range = 10.00- 23.91 | Range = 14.45- 36.93 | Range = 19.68- 31.10 |
| **Average** | 5.30 | 360.33 | 26.95 | 20.30 | 25.65 | 27.05 |
| **SD** | 0.30 | 134.80 | 15.79 | 9.05 | 11.24 | 6.39 |
| **SE** | 0.17 | 77.92 | 9.12 | 5.23 | 6.49 | 3.69 |

**Table H: Homocysteine and p53 expression pattern in Megaloblastic anemia cases (Normal range Males: 6 – 22µmol/L; Females: 3-18 µmol/L)**

| **Sample No.** | **Sex** | **Homocysteine**  **(µmol/L)** | **P53 expression (% of Total Cells)** | | | |
| --- | --- | --- | --- | --- | --- | --- |
|  | | | **Unstained** | **Low** | **Moderate** | **Heavy** |
| 33 | M | 28.30 | 6.67 | 16.78 | 29.10 | 47.43 |
| 43 | M | 30.00 | 4.23 | 18.41 | 26.66 | 50.68 |
| 45 | M | 29.10 | 22.77 | 13.05 | 15.44 | 48.72 |
| 46 | M | 46.00 | 4.55 | 14.08 | 28.29 | 53.06 |
| 54 | M | 68.90 | 24.39 | 10.80 | 21.06 | 43.74 |
| 55 | M | 26.20 | 21.03 | 13.61 | 20.13 | 45.21 |
| 56 | M | >80.00 | 17.00 | 6.00 | 10.53 | 66.55 |
| 58 | F | 78.00 | 5.85 | 13.00 | 21.13 | 60.01 |
| 60 | M | 13.50 (normal) | 11.38 | 22.00 | 22.77 | 43.83 |
| 61 | M | >80.00 | 5.69 | 12.84 | 20.46 | 61.00 |
| 63 | M | 21.50 (normal) | 10.58 | 20.13 | 25.59 | 43.68 |
| 67 | F | 17.80 (normal) | 19.39 | 23.91 | 25.58 | 31.10 |
| 72 | F | 25.60 | 4.33 | 23.28 | 28.08 | 44.29 |
| 73 | F | 24.00 | 3.13 | 21.36 | 27.63 | 48.00 |
| 76 | F | 55.80 | 13.32 | 13.00 | 22.81 | 51.00 |
| 79 | F | 15.00 (normal) | 14.14 | 18.85 | 31.87 | 35.12 |
| 82 | F | 11.20 (normal) | 20.34 | 14.00 | 23.57 | 42.18 |
| 83 | M | 48.80 | 25.89 | 12.70 | 15.79 | 45.60 |
| 84 | F | >80.00 | 1.08 | 8.38 | 21.38 | 69.14 |
| 89 | M | 72.60 | 16.27 | 9.74 | 13.44 | 60.69 |
| 90 | M | 80.00 | 17.32 | 9.56 | 15.00 | 57.70 |
| 91 | M | >80.00 | 0.90 | 8.52 | 13.04 | 77.52 |
| 92 | M | 24.90 | 16.63 | 25.22 | 25.00 | 33.09 |
| 93 | M | 52.50 | 17.20 | 13.51 | 21.08 | 48.19 |
| 94 | M | 17.80 (normal) | 26.28 | 13.40 | 16.19 | 44.11 |
| 95 | M | 79.60 | 15.00 | 4.17 | 9.79 | 71.02 |
| 96 | F | 62.50 | 10.94 | 6.35 | 15.62 | 67.06 |
| 97 | M | 22.60 | 23.25 | 10.09 | 14.76 | 52.00 |
| 98 | F | 40.50 | 25.05 | 9.00 | 17.17 | 48.80 |
| 100 | M | >80.00 | 5.33 | 5.66 | 15.30 | 73.68 |
| **N=30** |  | Normal HCys level – N=6 (20%)  High HCys level – N=24 (80%) | Range = 0.99 – 26.28 | Range = 4.17 – 23.91 | Range = 9.79 – 29.10 | Range = 31.10 – 77.52 |
| **Average** |  | 46.42 | 13.66 | 13.71 | 20.47 | 52.14 |
| **SD** |  | 25.81 | 8.03 | 5.75 | 5.91 | 11.93 |
| **SE** |  | 4.72 | 1.46 | 1.05 | 1.08 | 2.18 |

**Table I: Homocysteine and p53 expression pattern in non-megaloblastic anemia subjects (Normal range Males: 6-22µmol/L; Female: 3-18 µmol/L)**

| **Sample No.** | **Sex** | **Homocysteine**  **(µmol/L)** | **P53 expression (% of Total Cells)** | | | |
| --- | --- | --- | --- | --- | --- | --- |
|  | | | **Unstained** | **Low** | **Moderate** | **Heavy** |
| 42 | M | 11.80 | 66.87 | 15.19 | 10.73 | 7.19 |
| 44 | F | 7.00 | 72.94 | 8.75 | 9.85 | 8.45 |
| 47 | F | 11.00 | 75.52 | 16.00 | 5.48 | 2.97 |
| 48 | F | 6.00 | 55.30 | 23.00 | 13.46 | 8.30 |
| 49 | F | 7.10 | 49.14 | 24.41 | 16.64 | 9.79 |
| 50 | F | 4.40 | 61.88 | 20.09 | 11.00 | 7.00 |
| 51 | F | 6.00 | 43.47 | 37.43 | 12.56 | 6.52 |
| 52 | F | 7.60 | 95.28 | 2.46 | 1.21 | 1.03 |
| 53 | F | 3.90 | 50.67 | 27.28 | 13.55 | 8.47 |
| 57 | M | 9.50 | 96.18 | 2.12 | 1.13 | 0.56 |
| 59 | M | 10.30 | 36.07 | 29.42 | 24.44 | 10.05 |
| 62 | F | 17.10 | 41.00 | 28.14 | 20.11 | 10.77 |
| 64 | M | 13.90 | 55.84 | 20.00 | 15.37 | 8.85 |
| 65 | F | 2.50 (low) | 46.06 | 43.00 | 8.36 | 2.65 |
| 66 | F | 4.50 | 85.69 | 7.09 | 4.76 | 2.44 |
| 68 | F | 11.70 | 46.02 | 22.52 | 19.00 | 12.47 |
| 69 | M | 7.40 | 55.34 | 19.87 | 14.79 | 10.00 |
| 70 | M | 2.50 (low) | 57.53 | 23.61 | 12.43 | 6.41 |
| 71 | M | 2.50 (low) | 60.23 | 27.15 | 7.66 | 0.55 |
| 74 | M | 12.10 | 42.48 | 23.79 | 18.68 | 15.03 |
| 75 | F | 13.00 | 36.61 | 46.46 | 13.70 | 3.21 |
| 77 | F | 30.30 (high) | 46.80 | 30.91 | 15.35 | 6.92 |
| 78 | F | 18.90 (high) | 53.18 | 24.68 | 14.02 | 8.11 |
| 80 | M | 16.60 | 57.26 | 21.49 | 12.61 | 8.61 |
| 81 | F | 14.60 | 47.87 | 27.00 | 15.79 | 9.32 |
| 85 | F | 20.20 (high) | 28.71 | 33.86 | 22.69 | 14.70 |
| 86 | F | 25.10 (high) | 25.00 | 35.10 | 23.95 | 15.92 |
| 87 | M | 12.70 | 80.91 | 12.10 | 4.10 | 2.87 |
| 88 | M | 28.40 (high) | 23.30 | 37.00 | 24.27 | 15.42 |
| 99 | F | 20.70 (high) | 39.87 | 35.31 | 17.85 | 6.95 |
| **N=30** |  | Normal HCys level – N=24 (80%)  High HCys level – N=6 (20%) | Range = 23.30 – 96.18 | Range = 2.12 – 46.46 | Range = 1.13 – 24.44 | Range = 0.55 – 15.92 |
| **Average** |  | 11.97 | 54.43 | 24.17 | 13.51 | 7.71 |
| **SD** |  | 7.53 | 18.78 | 10.96 | 6.42 | 4.37 |
| **SE** |  | 1.37 | 3.43 | 2.00 | 1.17 | 0.79 |
